# Association between lactate/albumin ratio and 28-day all-cause mortality in ischemic stroke patients without reperfusion therapy: a retrospective analysis of the MIMIC-IV database

**DOI:** 10.3389/fneur.2023.1271391

**Published:** 2023-10-12

**Authors:** Yuan Zhong, Hao Sun, Hongzhuang Chen, Wenjuan Jing, Weiqiang Chen, Junqiang Ma

**Affiliations:** ^1^Department of Neurosurgery, First Affiliated Hospital of Shantou University Medical College, Shantou, Guangdong, China; ^2^Department of Critical Care Medicine, First Affiliated Hospital of Shantou University Medical College, Shantou, Guangdong, China; ^3^Department of Dermatology, First Affiliated Hospital of Shantou University Medical College, Shantou, Guangdong, China

**Keywords:** lactate/albumin ratio, ischemic stroke, all-cause mortality, 28-day, prognosis

## Abstract

**Objective:**

The lactate/albumin ratio (LAR) has been used as a novel prognostic indicator for aneurysmal subarachnoid hemorrhage, traumatic brain injury, sepsis, heart failure, and acute respiratory failure. However, its potential in predicting all-cause mortality in patients with ischemic stroke (IS) has not been evaluated. Therefore, this study aimed to elucidate the correlation between LAR and 28-day all-cause mortality in IS patients without reperfusion therapy.

**Methods:**

This retrospective cohort study used data from the Medical Information Mart for Intensive Care (MIMIC-IV) (v2.0) database. It included 568 IS adult patients admitted to the intensive care unit (ICU). The correlation between LAR and ICU 28-day all-cause mortality rate was analyzed using multiple COX regression analysis and Kaplan–Meier survival analysis. Restricted cubic spline (RCS) curves were used to assess the relationship between LAR and 28-day mortality. In addition, a subgroup analysis was performed to investigate the impact of other influencing factors on outcomes. The primary outcome was the ability of LAR to predict 28-day mortality in IS patients.

**Results:**

Among the 568 patients with IS, 370 survived (survival group) and 198 died (non-survival group) within 28 days of admission (mortality rate: 34.9%). A multivariate COX regression analysis indicated that LAR was an independent predictor of all-cause mortality within 28 days after admission for patients with IS (hazard ratio: 1.32; 95% confidence interval: 1.03–1.68; *P* = 0.025). We constructed a model that included LAR, age, race, sex, white blood cell count, Sequential Organ Failure Assessment (SOFA) score, and anion gap (AG) and established a prediction model with an area under the curve (AUC) value of 71.5% (95% confidence interval: 67.1%−75.8%). The optimal cutoff value of LAR that separated the survival group and the non-survival group based on the Youden index was 0.55. The Kaplan-Meier survival curves plotted using this critical value showed that patients with LAR ≥ 0.55 had a significantly higher 28-day all-cause mortality rate than patients with LAR < 0.55 (*P* = 0.0083).

**Conclusion:**

LAR can serve as an independent predictor of all-cause mortality within 28 days after admission for patients with IS.

## 1. Introduction

Ischemic stroke (IS) is a grave condition that affects the blood vessels in the brain and endangers the life of patients. It is the second most common cause of disability and death across the globe (1). Approximately 15% of IS patients die within 30 days (1). In recent years, the emergence of venous thrombolysis and endovascular thrombectomy has ushered us into a new era of IS treatment, wherein efficient reperfusion therapy is widely employed (2). However, numerous patients cannot be treated with reperfusion therapy in time, especially in developing countries. Therefore, there is an urgent need for simple and practical risk indicators that can inform the clinical management of IS patients without reperfusion therapy.

Lactate, a by-product of anaerobic metabolism, indicates the degree of tissue underperfusion and cellular oxygen deprivation (3). It can also forecast organ dysfunction and death in critically ill patients (4). Besides, it plays a crucial role in IS prognosis because it accumulates rapidly due to impaired diffusion of ischemic brain tissue, and its excess causes acidosis, which activates specific ion channels, leading to neurotoxic calcium accumulation and cytotoxic swelling (5). However, protein hydrolysis metabolism and metformin intake in patients with liver dysfunction or abnormalities can lead to abnormal lactate levels (6, 7). Therefore, relying solely on lactate levels for prediction may not guarantee reliable results.

Albumin is a vital protein that regulates blood osmotic pressure and influences the physiological function of the circulatory system. It also exhibits anti-inflammatory, antioxidant, and antithrombotic effects. However, serum albumin levels are influenced by kidney disease or nutritional status and therefore have limited value on their own in predicting IS outcomes (8, 9).

Some studies have explored the lactate/albumin ratio (LAR) as a potential predictor of acute pancreatitis, severe pneumonia, traumatic brain injury, and aneurysm subarachnoid hemorrhage (10–13). However, the link between LAR and mortality in IS patients remains unknown. Therefore, we obtained and analyzed data on IS patients admitted between 2008 and 2019 from the MIMIC-IV (v2.0) database. The current study aims to analyze the relationship between LAR and all-cause mortality within 28 days of admission in IS patients.

## 2. Methods

### 2.1. Data collection

We obtained our data from the MIMIC-IV (v2.0), a large-scale, open-source database created and maintained by the MIT Computational Physiology Laboratory (https://physionet.org/content/mimiciv/2.0/). This database contains the records of all the patients hospitalized at the Beth Israel Deaconess Medical Center (BIDMC) between 2008 and 2019. It provides comprehensive data, such as length of stay, laboratory results, medication administration, vital signs, etc., for each patient. The data was anonymized by replacing personal information with random codes to protect patient privacy, so we did not require patient consent or ethical approval. The MIMIC-IV (v2.0) database is available for download from the PhysioNet online platform (https://physionet.org/). To access the database, the second author of this study, Chen HongZhuang, completed the Collaborative Institutional Training Initiative (CITI) course and passed the exams on “Conflict of Interest” and “Data or Sample Only Research” (ID: 52748910). The research team was then authorized to use the database and extract data.

### 2.2. Population selection criteria

**Figure 1:**
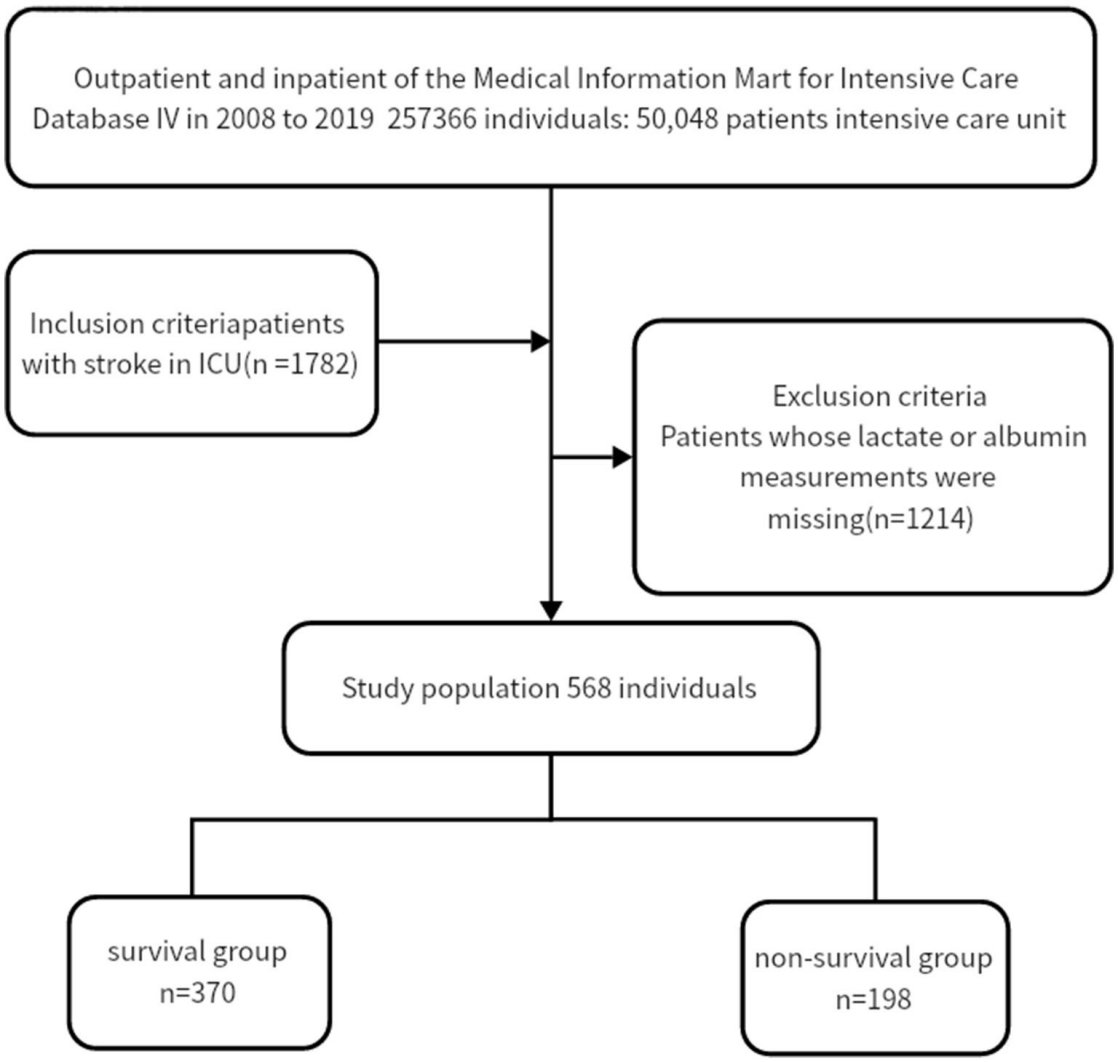
Flowchart of patient inclusion.

We selected patients from the MIMIC-IV database using the following criteria: (1) age above 18 years, and (2) IS diagnosis based on ICD-9 codes 433, 434, 436, 437.0, and 437.1 or ICD-10 codes I63 and I65 (Figure 1). We excluded patients who underwent reperfusion therapy and those without lactate or albumin measurements. If patients had multiple ICU admissions, we only used clinical data from the first ICU admission. Ultimately, 568 patients were included in this study.

### 2.3. Data extraction

We selected LAR as the primary variable of interest. We used the first blood lactate and serum albumin levels measured after admission to reduce the influence of subsequent treatments on these values. Potential confounders, such as demographics (age, sex, and race), vital signs (heart rate, systolic blood pressure, and diastolic blood pressure), comorbidities (myocardial infarction, congestive heart failure, peripheral vascular disease, dementia, chronic pulmonary disease, rheumatic disease, peptic ulcer disease, liver disease, diabetes, paraplegia, renal disease, AIDS, and hemorrhage), laboratory tests (red blood cell, white blood cell, and red blood cell distribution width, platelet, hemoglobin, and lymphocyte percentage, hematocrit, serum glucose level, anion gap, prothrombin time, and international normalized ratio), and sequential organ failure assessment (SOFA) scores were also extracted. Data extraction was performed using PostgreSQL (v13.7.1) and Navicate Premium (version 15) with structured query language. All the code for computing demographic features, laboratory tests, comorbidities, and severity scores were obtained from the GitHub website (GitHub - MIT-LCP/mimic-iv: Deprecated. For the latest MIMIC-IV code see: https://github.com/MIT-LCP/mimic-code).

### 2.4. Grouping and endpoint events

This study classified the patients into two groups: those who survived in the hospital for 28 days (survival group, *n* = 370) and those who died in the hospital within 28 days (non-survival group, *n* = 198). The primary outcome of interest was all-cause mortality within 28 days of admission.

### 2.5. Management of missing data and outliers

Variables with more than 15% missing values, such as blood cholesterol, triglycerides, high-density lipoprotein, low-density lipoprotein, and C-reactive protein, were omitted to reduce bias. For variables with <15% missing values (lymphocyte, monocyte, and neutrophil counts), multiple imputation was applied to choose the best possible data set to impute the missing values (14).

### 2.6. Statistical analysis

The Kolmogorov-Smirnov test evaluated the normality of continuous variables. Continuous variables were reported as mean ± SD for normal distributions, median (IQR) for skewed distributions, and frequencies (%) for categorical variables. In the baseline characteristics analysis, continuous variables were compared using T-test or one-way ANOVA, while categorical variables were compared using Pearson's χ2 test and Fisher's test. A univariate COX regression analysis identified potential risk factors and a multivariate COX regression analysis determined the independent risk factors for in-hospital mortality with p-values below 0.05. A receiver operating characteristic (ROC) analysis assessed the model's predictive performance for 28-day in-hospital mortality by calculating the area under the curve (AUC), sensitivity, and specificity of the models. The Youden index was utilized to determine the optimal cutoff value.

Restricted cubic spline (RCS) analysis was employed to depict the non-linear relationship between LAR and 28-day all-cause mortality in IS subjects.

Kaplan–Meier curves were used to observe the relationship between LAR and mortality rate in IS patients. Subgroup analysis examined the effect of LAR on different characteristics, such as age, sex, race, SOFA scores, white blood cell (WBC) count, anion gap, ventilation status, and cerebral hemorrhage, and their p-values for interactions were also tested. All analyses were performed with free statistical software version 1.6 and R 4.1.3 (R Foundation for Statistical Computing, Vienna, Austria). *P* < 0.05 from two-tailed tests indicated statistical significance.

## 3. Results

### 3.1. Baseline demographic and clinical characteristics

Table 1 shows the baseline characteristics of the survival and non-survival groups. Of the 568 patients who met the inclusion criteria, 309 (55.4%) were men and the median age was 67.8 (52.1, 83.5) years. The 28-day mortality rate was 34.9%. Non-survivors were older (*P* < 0.01) and had lower Glasgow Coma Scale scores, lower albumin levels (*P* < 0.05), higher SOFA scores, higher LAR [0.9 (0, 1.8) *vs*. 0.7 (0.1, 1.3), *P* = 0.002], and higher lactate levels (*P* < 0.05) than survivors. The other covariates were not significantly different between the groups (*P* > 0.05).

**Table 1:** Baseline characteristics between survivors(0) and non-survivors(1).

**Variables**	**Total (*n =* 568)**	**0 (*n =* 370)**	**1 (*n =* 198)**
Sex: male, *n* (%)	309 (54.4)	199 (53.8)	110 (55.6)
Age, mean ± SD	67.8 ± 15.7	66.1 ± 16.0	71.0 ± 14.5
**Ethnicity**, ***n*** **(%)**			
1 White	365 (64.3)	251 (67.8)	114 (57.6)
2 Black	71 (12.5)	47 (12.7)	24 (12.1)
3 Other	64 (11.3)	47 (12.7)	17 (8.6)
4 Unknown	68 (12.0)	25 (6.8)	43 (21.7)
SBP, mean ± SD	129.4 ± 27.8	129.4 ± 27.6	129.3 ± 28.3
DBP, mean ± SD	70.9 ± 20.0	70.5 ± 20.0	71.7 ± 20.2
SOFA, mean ± SD	7.4 ± 3.6	7.1 ± 3.4	7.9 ± 4.0
GCS, mean ± SD	9.1 ± 4.2	9.6 ± 4.1	8.1 ± 4.3
WBC, mean ± SD	13.1 ± 7.1	12.4 ± 6.6	14.4 ± 7.8
Neutrophils, mean ± SD	937.5 ± 735.7	904.9 ± 718.5	994.3 ± 763.5
Lymphocytes, mean ± SD	121.0 ± 208.1	109.8 ± 93.7	140.5 ± 321.5
Monocytes, mean ± SD	50.2 ± 61.2	47.1 ± 47.1	55.5 ± 79.9
Platelets, mean ± SD	229.3 ± 124.3	231.1 ± 131.0	225.8 ± 110.9
Hemoglobin, mean ± SD	11.5 ± 2.6	11.4 ± 2.6	11.6 ± 2.7
Glucose, mean ± SD	172.3 ± 99.9	171.0 ± 104.5	174.7 ± 90.9
Creatinine, mean ± SD	1.6 ± 1.5	1.6 ± 1.6	1.6 ± 1.4
Anion gap, mean ± SD	16.4 ± 5.1	15.8 ± 4.7	17.6 ± 5.6
PT, mean ± SD	17.4 ± 14.2	17.3 ± 13.8	17.5 ± 15.0
PTT, mean ± SD	36.4 ± 21.5	35.9 ± 20.3	37.2 ± 23.7
Phosphate, mean ± SD	3.8 ± 1.5	3.8 ± 1.4	3.9 ± 1.6
Lactate, mean ± SD	2.3 ± 2.0	2.1 ± 1.6	2.6 ± 2.5
Albumin, mean ± SD	3.2 ± 0.7	3.2 ± 0.7	3.1 ± 0.6
LAR, mean ± SD	0.8 ± 0.7	0.7 ± 0.6	0.9 ± 0.9
Myocardial infarct, *n* (%)	95 (16.7)	64 (17.3)	31 (15.7)
Congestive heart failure, *n* (%)	140 (24.6)	85 (23)	55 (27.8)
Peripheral vascular disease, *n* (%)	102 (18.0)	66 (17.8)	36 (18.2)
Cerebrovascular disease, *n* (%)	568 (100.0)	370 (100)	198 (100)
Dementia, *n* (%)	12 (2.1)	6 (1.6)	6 (3)
Chronic pulmonary disease, *n* (%)	129 (22.7)	91 (24.6)	38 (19.2)
Rheumatic disease, *n* (%)	15 (2.6)	10 (2.7)	5 (2.5)
Peptic ulcer disease, *n* (%)	12 (2.1)	7 (1.9)	5 (2.5)
Mild liver disease, *n* (%)	67 (11.8)	45 (12.2)	22 (11.1)
Severe liver disease, *n* (%)	16 (2.8)	11 (3)	5 (2.5)
Diabetes, *n* (%)	214 (37.7)	141 (38.1)	73 (36.9)
Paraplegia, *n* (%)	158 (27.8)	101 (27.3)	57 (28.8)
Renal disease, *n* (%)	129 (22.7)	84 (22.7)	45 (22.7)
Aids, *n* (%)	4 (0.7)	1 (0.3)	3 (1.5)
Charlson comorbidity index,	6.9 ± 2.7	6.7 ± 2.6	7.3 ± 2.8
Hemorrhage, *n* (%)	84	57	27
Atrial fibrillation, *n* (%)	254	162	92

DBP, Diastolic Blood Pressure; GCS, Glasgow coma scale; LAR, lactate-to-albumin ratio; SBP, Systolic Blood Pressure; SOFA, sequential organ failure assessment; WBC, white blood cell count.

### 3.2. LAR is an independent risk factor for all-cause mortality within 28 days of hospital admission

Unadjusted LAR showed a significant association with all-cause mortality within 28 days of admission [hazard ratio (HR), 1.45; 95% confidence interval (CI), 1.15–1.83; *P* = 0.002] according to the results of the univariate COX regression analysis (Table 2). In the multivariate COX regression analysis (Table 3), LAR remained significantly associated with higher in-hospital 28-day all-cause mortality after adjusting for potential confounding factors such as age, sex, and race (HR, 1.55; 95% CI, 1.23–1.96; *P* < 0.002) in Model 1. Moreover, in Model 2, which included additional adjustments for WBC count, anion gap, and SOFA score, LAR remained an independent predictor of increased mortality risk (HR, 1.32; 95% CI, 1.03–1.68; *P* = 0.025).

**Table 2:** Univariate Cox regression models evaluating the association between LAR and 28-day all-cause mortality with IS.

**Item**	**HR (95%CI)**	***P* (Wald's test)**
Sex	1.05 (0.79,1.39)	0.758
Age	1.02 (1.01,1.03)	0.001
**Ethnicity: ref**. = **1**		
2	1.08 (0.7,1.68)	0.731
3	0.84 (0.5,1.39)	0.49
4	2.6 (1.83,3.69)	< 0.001
SBP	1.0001 (0.9951, 1.0052)	0.957
DBP	1.0024 (0.9956, 1.0092)	0.485
SOFA	1.05 (1.01,1.09)	0.022
GCS	0.93 (0.9,0.97)	< 0.001
WBC	1.03 (1.01,1.05)	< 0.001
Neutrophils	1.0002 (1, 1.0004)	0.104
Lymphocytes	1.0005 (1, 1.0011)	0.042
Monocytes	1.0024 (1.0003, 1.0045)	0.026
Platelets	0.9998 (0.9987, 1.0009)	0.719
Hemoglobin	1.03 (0.98,1.09)	0.215
Glucose	1.0004 (0.9991, 1.0017)	0.564
Creatinine	1.01 (0.93,1.11)	0.752
Anion gap	1.05 (1.02,1.07)	< 0.001
Sodium	1.03 (1.01,1.05)	0.011
Potassium	1.11 (0.96,1.28)	0.168
PT	0.9999 (0.9902, 1.0096)	0.982
PTT	1.0021 (0.9959, 1.0083)	0.514
Phosphate	1.08 (0.99,1.19)	0.099
Lactate	1.11 (1.05,1.18)	< 0.001
Albumin	0.86 (0.7,1.05)	0.138
LAR	1.45 (1.15,1.83)	0.002
Myocardial infarction: 1 vs. 0	0.89 (0.61,1.31)	0.568
Congestive heart failure: 1 vs. 0	1.21 (0.88,1.65)	0.236
Peripheral vascular disease: 1 vs. 0	1.04 (0.73,1.5)	0.822
Dementia: 1 vs. 0	1.53 (0.68,3.46)	0.303
Chronic pulmonary disease: 1 vs. 0	0.76 (0.54,1.09)	0.132
Rheumatic disease: 1 vs. 0	0.9 (0.37,2.18)	0.813
Peptic ulcer disease: 1 vs. 0	1.21 (0.5,2.94)	0.676
Mild liver disease: 1 vs. 0	0.87 (0.56,1.36)	0.549
Severe liver disease: 1 vs. 0	0.8 (0.33,1.93)	0.613
Diabetes: 1 vs. 0	0.94 (0.71,1.26)	0.692
Paraplegia: 1 vs 0	1.07 (0.78,1.45)	0.681
Renal disease: 1 vs. 0	0.9963 (0.7146, 1.3891)	0.983
Aids: 1 vs. 0	3.39 (1.08,10.61)	0.036
Charlson comorbidity index	1.06 (1.01,1.11)	0.026
Hemorrhage: 1 vs. 0	0.89 (0.59,1.33)	0.556
Atrial fibrillation: 1 vs. 0	1.08 (0.82,1.43)	0.573

DBP, Diastolic Blood Pressure; GCS, Glasgow coma scale; LAR, lactate-to-albumin ratio; SBP, Systolic Blood Pressure; SOFA, sequential organ failure assessment; WBC, white blood cell count.

**Table 3:** Multivariable Cox regression models evaluating the association between LAR and 28-day all-cause mortality with IS.

**Variable**	**Crude. HR_95CI**	**Crude_*p*-value**	**Adj. HR_95CI**	**Adj. _*p*-value**
**Model 1**				
LAR	1.45 (1.15~1.83)	0.002	1.55 (1.23~1.96)	0.001
Sex: male	1.05 (0.79~1.39)	0.758	1.18 (0.88~1.58)	0.267
Age	1.02 (1.01~1.03)	0.001	1.02 (1.01~1.03)	0.001
Ethnicity 1	Ref			
Ethnicity 2	1.09 (0.7~1.7)	0.687	1.13 (0.72~1.76)	0.601
Ethnicity 3	0.85 (0.51~1.41)	0.524	0.93 (0.55~1.55)	0.773
Ethnicity 4	2.55 (1.78~3.65)	0.001	3.01 (2.09~4.33)	0.001
**Model 2**				
LAR	1.45 (1.15~1.83)	0.002	1.32 (1.03~1.68)	0.025
Sex: male	1.05 (0.79~1.39)	0.758	1.18 (0.88~1.58)	0.257
Age	1.02 (1.01~1.03)	0.001	1.02 (1.01~1.03)	< 0.001
Ethnicity 1	Ref			
Ethnicity 2	1.09 (0.7~1.7)	0.687	1.19 (0.76~1.88)	0.452
Ethnicity 3	0.85 (0.51~1.41)	0.524	0.94 (0.56~1.59)	0.821
Ethnicity 4	2.55 (1.78~3.65)	< 0.001	3.01 (2.08~4.35)	< 0.001
WBC	1.03 (1.01~1.05)	< 0.001	1.03 (1.01~1.05)	0.002
Anion gap	1.05 (1.02~1.07)	0.001	1.04 (1.01~1.07)	0.007
SOFA	1.05 (1.01~1.09)	0.022	1.03 (0.99~1.07)	0.2

Ethnicity 1, Wihte; Ethnicity 2, Black; Ethnicity 3, Others; Ethnicity 4, Unknown; LAR, lactate-to-albumin ratio; SOFA, sequential organ failure assessment; WBC, white blood cell count.

### 3.3. ROC curve analysis, RCS curves, and Kaplan–Meier curve

**Figure 2:**
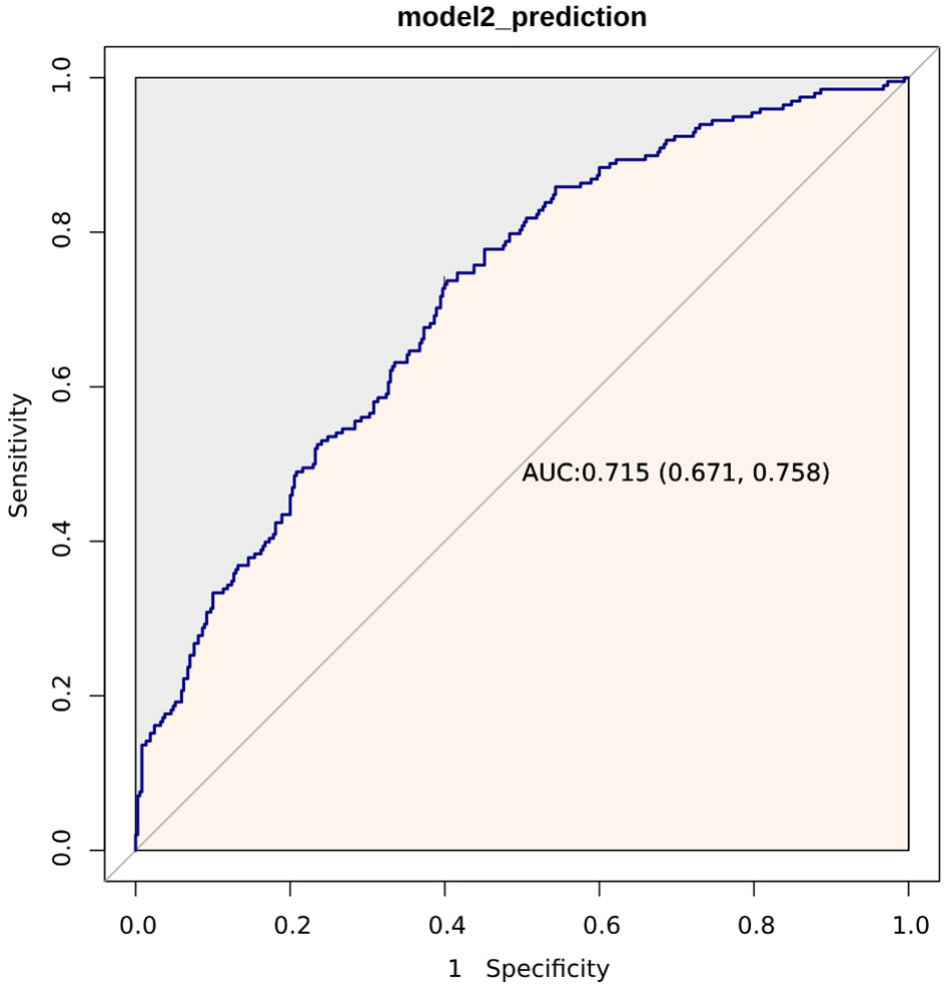
ROC curves of Model 2 for predicting 28-day mortality. Model 2 includes WBC count, anion gap, SOFA score, and LAR.

**Figure 3:**
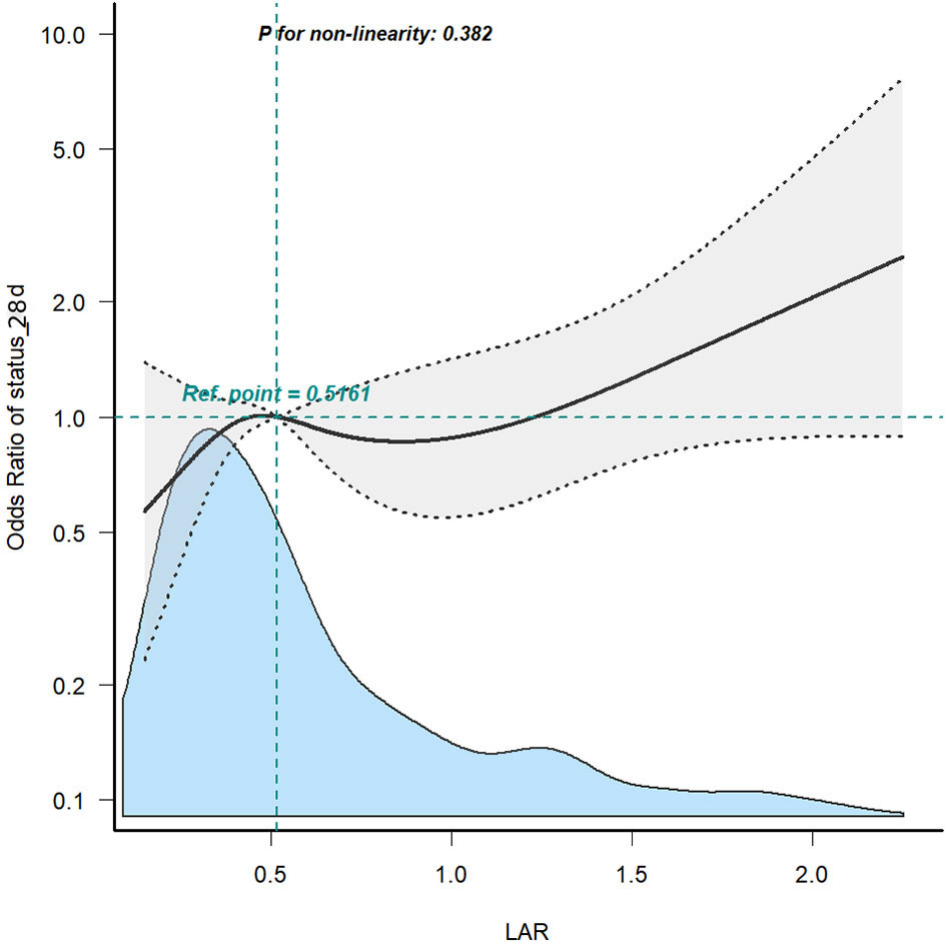
RCS curve for the LAR. Shaded ribbons denote 95% confidence intervals.

**Figure 4:**
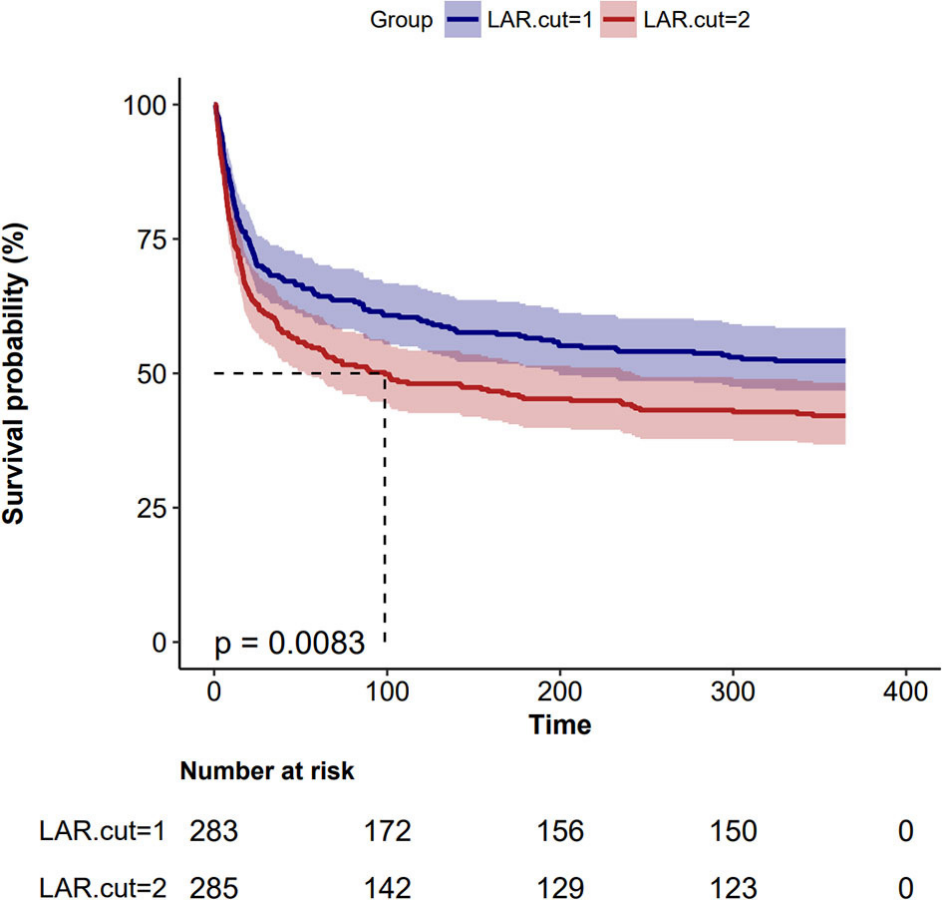
Kaplan–Meier survival analysis curves for 28-day all-cause mortality.

Figure 2 displays the ROC curves of Model 2 plotted for predicting all-cause mortality within 28 days after admission of IS patients, and the AUC of the model was 71.5% (95% CI: 67.1%−75.8%). Additionally, Model 2 had a sensitivity of 75.13% and a specificity of 58.81%. Based on the Youden index, we selected the optimal threshold value to divide the IS patients into a high LAR group (LAR ≥ 0.55, *n* = 283) and a low LAR group (LAR < 0.55, *n* = 285). An RCS analysis was employed to assess the non-linear relationship between LAR and 28-day mortality in IS subjects (Figure 3). The Kaplan–Meier survival curves (Figure 4) show that the mortality rate of the high LAR group was significantly higher than that of the low LAR group (*P* = 0.0083).

### 3.4. Subgroup analysis and forest plots

**Figure 5:**
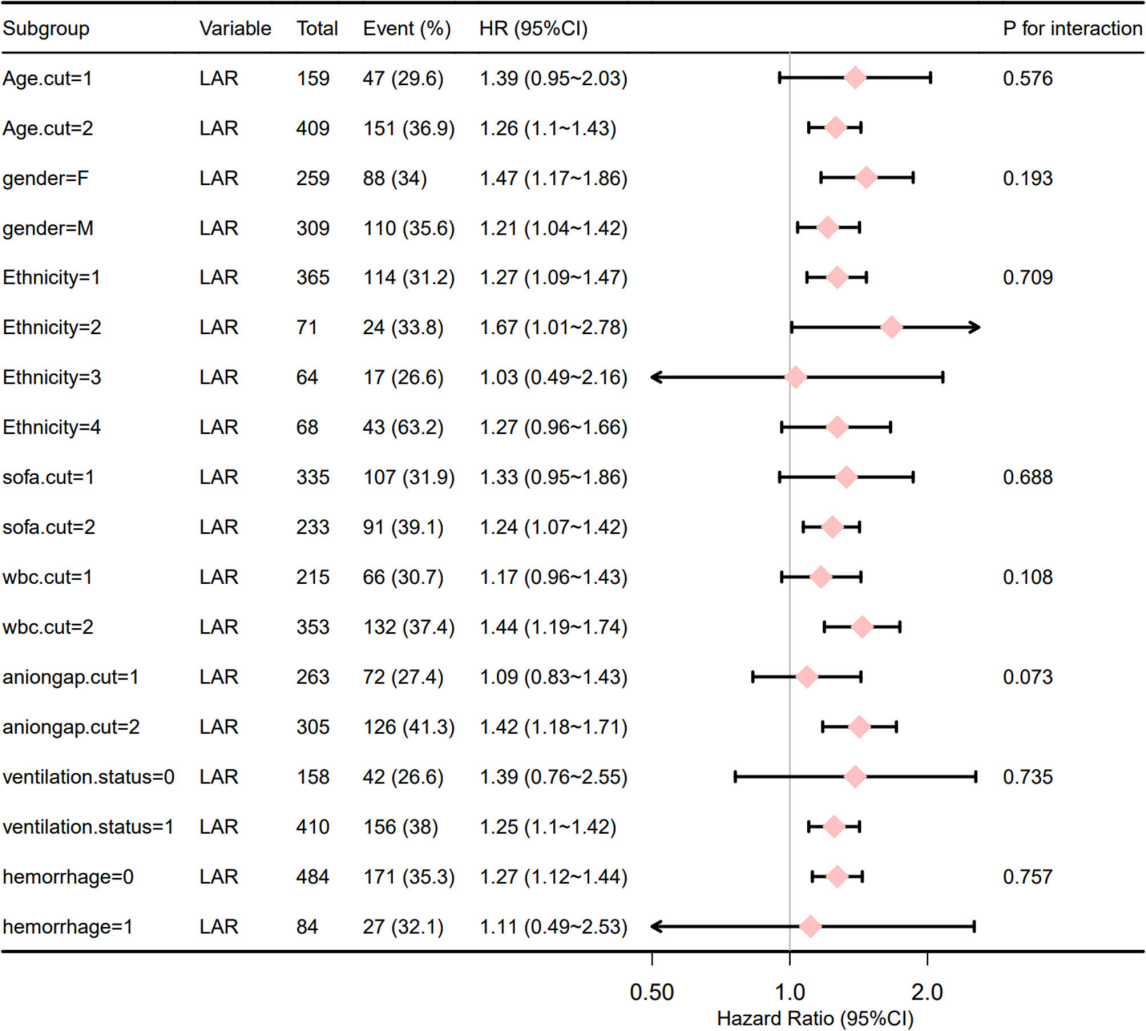
Forest plot for the subgroup analysis of the relationship between hospital mortality and LAR. Age.cut1, age < 67.8; Age.cut2, age > 67.8; Ethnicity1, White; Ethnicity2, Black; Ethnicity3, Other; Ethnicity4, Unknown; Sofa.cut1, sofa < 7; Sofa.cut2, sofa > 7; Wbc.cut1, WBC count < 13.1; Wbc.cut2, WBC count ≥ 13.1; aniogap.cut1, aniogap < 16.4; aniogap.cut2, aniogap > 16.4; for ventilation.status and hemorrhage, 0 means that the value does not exist and 1 means that it exists.

Figure 5 shows that the correlation between LAR and all-cause mortality within 28 days of admission of IS patients was stable across subgroups. The forest plot from the stratified analysis was performed for age, sex, race, SOFA score, WBC count, anion gap, ventilation status, and hemorrhagic transformation of cerebral infarction and showed that LAR had no significant interaction with each subgroup (interaction P: 0.073–0.735). These results prove that LAR was an independent prognostic factor.

## 4. Discussion

This is the first study examining the role of LAR in IS patients. The results of this retrospective study demonstrated that the LAR was an independent factor for all-cause mortality in IS patients without reperfusion therapy within 28 days of hospital admission. Our study included 568 patients from the MIMIC-IV (2.0) database. We conducted COX regression analysis to determine the independent predictive factors for 28-day mortality before and after adjusting for confounding factors. We found that LAR was consistently identified as an independent predictor of 28-day mortality. Additionally, we identified an optimal cutoff point (0.55), allowing us to construct a Kaplan–Meier curve and demonstrate that LAR effectively differentiated patients who died within 28 days. Furthermore, we adjusted for all confounding factors and created a forest plot, which showed that LAR remained a stable indicator unaffected by other variables. Therefore, LAR is reliable for predicting the 28-day mortality of IS patients and can be used as a novel clinical biomarker.

Lactate is an important indicator of tissue oxygenation, blood perfusion, and metabolism in the body. Hypoxia-induced acidosis in brain tissue is a sensitive indicator of brain injury (5). Lactate is a biomarker of ischemia produced by anaerobic glycolysis (15). Sakal et al. found that hyperlactatemia was correlated with increased mortality at 1, 3, and 12 months in IS patients (16, 17). However, interpreting serum lactate levels is indeed complex. For example, patients with liver disease may have abnormal lactate metabolism. Under hypoxic conditions, lactate production may also increase. Some drugs, such as salbutamol and metformin, can also elevate lactate levels. In addition, some critically ill patients may have lower lactate levels in venous blood, which reduces the reliability of lactate levels alone in predicting patient outcomes (18).

Serum albumin is associated with the outcome of IS (19). Serum albumin extravasation into the ischemic brain may provide neuroprotection by limiting metal-catalyzed oxidative stress (20). Gao et al. found that a decline in serum albumin levels after 90 days of acute large vessel occlusive stroke was independently associated with poor prognosis (21). Dziedzic et al. and Babu et al. suggested that higher serum albumin levels in acute stroke patients could reduce the risk of adverse outcomes (22, 23).

A meta-analysis (24) including 13,618 patients with acute IS or transient ischemic attack concluded that low serum albumin levels could predict adverse functional outcomes and mortality in patients with these diseases. However, different albumin detection methods in different studies may have biased the results. In addition, serum albumin levels are influenced by underlying diseases, nutritional status, and inflammation, which may limit its prognostic value as a single measurement. In the present study, we took the ratio of blood lactate to serum albumin, reducing the influence of a single factor on the regulatory mechanism by causing inverse changes through two different mechanisms, thus more accurately predicting the outcome for IS patients.

Recently, researchers explored the predictive value of LAR in the prognosis of neurosurgical diseases. For example, Wang et al.'s cohort study (12) on the mortality of patients with moderate to severe traumatic brain injury showed that non-survivors had higher LAR than survivors (1.09 *vs*. 0.53, *P* < 0.001), which was close to our results. Zhang et al. (13) established a prediction model for in-hospital mortality of patients with spontaneous subarachnoid hemorrhage. Independent predictors included age, LAR, anion gap, and Acute Physiology Score III, which was similar to our prediction model. Their results showed that LAR was closely related to increased in-hospital mortality of patients with spontaneous subarachnoid hemorrhage. However, studies using LAR to predict the outcome of ischemic stroke patients have yet to be reported.

Our results also confirmed previous research findings on the association between WBC count at admission and the prognosis of patients with ischemic stroke. Zheng et al. demonstrated that elevated WBC counts are correlated with stroke severity and adverse major and minor outcomes within a 3-month period (25). Furlan et al. reported that with each increase of 1 × 10(-9)/l in WBC count, there is a proportional rise in stroke severity, degree of disability at discharge, and 30-day mortality (26).

In addition to WBC count, our study also investigated the association between the anion gap and the prognosis of patients with ischemic stroke. Consistent with prior studies, Wang et al. observed a significant association between elevated AG values and increased all-cause mortality rates at 1 year, 4 years, and overall in patients with ischemic stroke who received rtPA treatment (27). Furthermore, Liu et al. demonstrated that high AG is an independent risk factor for all-cause mortality at 30 days, 60 days, and 180 days in patients with ischemic stroke (28). These collective study findings suggest that AG has the potential to serve as a biomarker for predicting the prognosis of patients with ischemic stroke.

Patients with IS admitted to the ICU have a higher mortality rate than other patients with IS. This may explain why the mortality rate of the patients included in our study was higher than the overall mortality rate of IS patients. In a study involving 370,386 ICU patients [including 7,046 (1.9%) stroke patients, with 4,072 having IS and 2,974 having intracerebral hemorrhage] (29), the short-term mortality rate of stroke patients admitted to the ICU was higher, with a 30-day mortality rate of 31% for IS patients, which is similar to our study.

In our study, LAR could be used as an independent predictor of 28-day all-cause mortality in IS patients without reperfusion therapy; it yielded a more accurate prognosis than blood lactate or serum albumin alone. This will provide medical workers with a better tool for clinic planning for poor patient outcomes. Further validation of LAR as a readily available and objective biomarker is still needed in large-scale multicenter prospective studies.

Our study has some limitations. First, it is a single-center retrospective cohort study, which cannot elucidate the relationship between LAR and IS as prospective studies do, to the extent that our findings need more persuasive power. Second, the drugs and hospital medical care, which may affect the LAR of patients with IS, were not recorded, which might bias our results. Lastly, although potential confounding factors such as myocardial infarction, congestive heart failure, peripheral vascular disease, dementia, liver disease, and hemorrhage were not significantly present in our results, they should be considered and potentially excluded in future prospective studies.

## 5. Conclusion

LAR can serve as an independent predictor of all-cause mortality within 28 days after admission for IS patients without reperfusion therapy.

## Data availability statement

The data analyzed in this study was obtained from the Medical Information Mart for Intensive Care IV (MIMIC-IV) database, the following licenses/restrictions apply: To access the files, users must be credentialed users, complete the required training (CITI Data or Specimens Only Research) and sign the data use agreement for the project. Requests to access these datasets should be directed to PhysioNet, https://physionet.org/, doi: 10.13026/6mm1-ek67.

## Ethics statement

Ethical review and approval was not required for the study on human participants in accordance with the local legislation and institutional requirements. Written informed consent from the patients/participants or patients/participants' legal guardian/next of kin was not required to participate in this study in accordance with the national legislation and the institutional requirements.

## Author contributions

YZ: Conceptualization, Data curation, Formal analysis, Funding acquisition, Investigation, Methodology, Project administration, Resources, Software, Supervision, Validation, Visualization, Writing—original draft, Writing—review and editing. HS: Conceptualization, Data curation, Formal analysis, Funding acquisition, Investigation, Methodology, Project administration, Resources, Software, Supervision, Validation, Visualization, Writing—original draft, Writing—review and editing. HC: Conceptualization, Data curation, Investigation, Methodology, Software, Supervision, Writing—review and editing. WJ: Data curation, Formal analysis, Methodology, Project administration, Supervision, Validation, Writing—review and editing. WC: Conceptualization, Data curation, Formal analysis, Funding acquisition, Investigation, Methodology, Project administration, Resources, Software, Supervision, Validation, Visualization, Writing—original draft, Writing—review and editing. JM: Conceptualization, Data curation, Formal analysis, Funding acquisition, Investigation, Methodology, Project administration, Resources, Software, Supervision, Validation, Visualization, Writing—original draft, Writing—review and editing.
