# Corrigendum: Association between lactate/albumin ratio and 28-day all-cause mortality in ischemic stroke patients without reperfusion therapy: a retrospective analysis of the MIMIC-IV database

**DOI:** 10.3389/fneur.2024.1501758

**Published:** 2024-10-15

**Authors:** Yuan Zhong, Hao Sun, Hongzhuang Chen, Wenjuan Jing, Weiqiang Chen, Junqiang Ma

**Affiliations:** ^1^Department of Neurosurgery, First Affiliated Hospital of Shantou University Medical College, Shantou, Guangdong, China; ^2^Department of Critical Care Medicine, First Affiliated Hospital of Shantou University Medical College, Shantou, Guangdong, China; ^3^Department of Dermatology, First Affiliated Hospital of Shantou University Medical College, Shantou, Guangdong, China

**Keywords:** lactate/albumin ratio, ischemic stroke, all-cause mortality, 28-day, prognosis

In the published article, there was an error. In the section **Methods** *2.2. Population selection criteria*, paragraph 2, it previously stated:

“We selected patients from the MIMIC-IV database using the following criteria: (1) age above 18 years, and (2) IS diagnosis based on ICD-9 codes 433, 434, 436, 437.0, and 437.1 or I10, I63, and I65 (Figure 1).”

The corrected sentence appears below:

“We selected patients from the MIMIC-IV database using the following criteria: (1) age above 18 years, and (2) IS diagnosis based on ICD-9 codes 433, 434, 436, 437.0, and 437.1 or ICD-10 codes I63 and I65 (Figure 1).”

The authors apologize for this error and state that this does not change the scientific conclusions of the article in any way. The original article has been updated.

